# Understanding the domain of driving distraction with knowledge graphs

**DOI:** 10.1371/journal.pone.0278822

**Published:** 2022-12-09

**Authors:** Wenxia Xu, Lei Feng, Jun Ma

**Affiliations:** 1 School of Automotive Studies, Tongji University, Shanghai, China; 2 College of Design and Innovation, Tongji University, Shanghai, China; Zhejiang Univeristy, CHINA

## Abstract

This paper aims to provide insight into the driving distraction domain systematically on the basis of scientific knowledge graphs. For this purpose, 3,790 documents were taken into consideration after retrieving from *Web of Science Core Collection* and screening, and two types of knowledge graphs were constructed to demonstrate bibliometric information and domain-specific research content respectively. In terms of bibliometric analysis, the evolution of publication and citation numbers reveals the accelerated development of this domain, and trends of multidisciplinary and global participation could be identified according to knowledge graphs from Vosviewer. In terms of research content analysis, a new framework consisting of five dimensions was clarified, including “objective factors”, “human factors”, “research methods”, “data” and “data science”. The main entities of this domain were identified and relations between entities were extracted using Natural Language Processing methods with Python 3.9. In addition to the knowledge graph composed of all the keywords and relationships, entities and relations under each dimension were visualized, and relations between relevant dimensions were demonstrated in the form of heat maps. Furthermore, the trend and significance of driving distraction research were discussed, and special attention was given to future directions of this domain.

## Introduction

Road traffic crash was ranked the eighth leading cause of death by World Health Organization (WHO), claiming more than 1.35 million lives and causing up to 50 million injuries globally each year [1]. Humans are the weakest part of the road traffic system and it was estimated by the US National Highway Traffic Safety Administration (NHTSA) that 94% of the crashes could be attributed to drivers [2]. Among all the driver-related reasons, driving distraction is not to be ignored, which led to 9% of fatal crashes and 15% of injury crashes across the USA in 2019 [3]. Due to the severe consequences caused by driving distraction, scientific research into this issue is of great practical importance.

In the academic world, driving distraction or driver distraction is commonly defined as *“the diversion of attention away from activities critical for safe driving toward a competing activity*, *which may result in insufficient or no attention to activities critical for safe driving”* [4]. Several studies have summarized certain aspects of driving distraction. For example, the distracting effect of telephones [5], the impact of roadside billboards [6] and young drivers’ distraction problems [7] have been discussed thoroughly. And there have also been reviews concentrating on certain research methods like naturalistic driving study [8], on certain distraction measurements like eye movement [9] and on certain data processing ways like the application of computer vision in distraction recognition [10]. Meanwhile, more general themes that contain driving distraction have been systematically addressed, such as driver understanding and modeling [11], contributors of road accidents [12], as well as road safety [13, 14]. However, there exists a gap of a comprehensive overview concerning the driving distraction domain exactly. Thus, this study aims to complete this task.

To achieve the goal of providing a systematic understanding of the driving distraction domain, the scientific tool of knowledge graphs is adopted, which normally appear as multi-relational graphs composed of nodes and edges [15]. While the nodes represent entities or significant concepts, the edges linking nodes serve the purpose of exhibiting relations between entities. And two methods are employed for graph construction:

- The first is the bibliometric analysis of existing literature. In addition to traditional bibliometric methods such as yearly quantitative distribution, software tool specializing in automatic text-mining and visualization of bibliometrics is adopted in this work. Similar approaches have been extensively applied to social science [16, 17], medicine [18], workplace safety [19], safety climate [20] and road safety [13, 14]. One of the efforts of this paper is to analyze the driving distraction domain using bibliometrics-based knowledge graphs.
- The second is the establishment of a framework specially for the research content analysis of this domain and the construction of knowledge graphs under this framework through text mining. Mature text-mining and visualization tools designed for general use have been proved to be effective for bibliometric analysis, as mentioned in the first method. However, when it comes to content analysis, although general tools could produce keyword co-occurrence maps, which present the most frequent keywords and keywords appearing most frequently in the same documents [21], the limitations of embedded algorithms and graphical user interfaces manifest. For one thing, since the embedded algorithms are unable to extract phrases as minimum meaning units like “deep learning” in sentences of titles and abstracts, only listed keywords are generally utilized to form the map, leading to serious information loss. For another, since all the keywords are just symbols for the software and the semantic information is lost, it is impossible for words in different dimensions to be categorized, which may obscure understanding. One solution of the algorithms’ limitations could be the incorporation of human knowledge. As a matter of fact, constructing specialized knowledge graphs with the combination of human knowledge and objective algorithms has become a trend, and similar works have been completed in fields like arts and humanities [22], medicine [23], disease [24], public security [25] and energy [26]. Therefore, instead of obtaining graphs automatically with general tools, this study aims to go further to develop a specific framework specially for the driving distraction domain and create knowledge graphs on this basis. This paper is the first of its kind in driving distraction or even the more general road safety field.

## Materials and methods

### Data source

The *Web of Science Core Collection* was retrieved using (“drive” OR “driving” OR “driver*”) AND (“distract*” OR “inattention*” OR “non driving task*” OR “nondriving task*” OR “multi task*” OR “multitask*” OR “dual task*” OR “secondary task*”) in the *Topic* field to obtain the original data for this study. Document types were restricted to articles and review articles, and publication years were limited to 1990–2022. Then articles within categories that are irrelevant to this study, such as *Cell Biology*, *Dentistry Oral Surgery Medicine* and *Nanoscience Nanotechnology*, were excluded through manual check. The retrieval was updated on February 6th, 2022 and yielded 3,790 documents altogether. All the contents of *Full Record and Cited References* were exported to plain text file for subsequent analysis.

### Bibliometric analysis

Bibliometric analysis was conducted in terms of five aspects: yearly quantitative distribution of literature, major publication sources, productive and influential countries/regions, productive and influential organizations, as well as productive and influential researchers.

For the first aspect, yearly publications and citations were counted. As baseline reference, number changes of driving related articles were considered. To gain the number of driving related studies, the *Web of Science Core Collection* was retrieved using (“drive” OR “driving” OR “driver*”) in the *Topic* field, and document types were also restricted to articles and review articles. The publication numbers of 1990, 2000, 2010 and 2021 were recorded, and then growth rates were calculated using these numbers. Although checking all these documents manually is impossible due to the large quantity, the growth rates serve as estimations of relative number changes. After that, these growth rates were compared to those of driving distraction publications over the same period. Then a statistical graph regarding the publication and citation numbers of the driving distraction domain was drawn for explanation and three development stages were divided on this basis.

As regards to the latter four parts, the software tool VOSviewer (visualization of similarities viewer) developed by van Eck NJ and Waltman L was employed [27], and thresholds of document and citation numbers were set in the software to obtain the most productive and influential publication sources, countries/regions, organizations and researchers for visualization respectively. The composition of the four graphs is in accordance with knowledge graphs’ normal appearance of multi-relational graphs composed of entities (nodes) and relations (edges) [15]. The node size implies the document number, while the node color implies the average publication year of the documents by a source, a country/region, an organization or a researcher, with a color bar at the bottom right of each graph for explanation. The edge weights in all the four graphs indicate the bibliographic coupling relationships, namely the number of cited references that two entities have in common.

### Research content analysis

**Fig 1:**
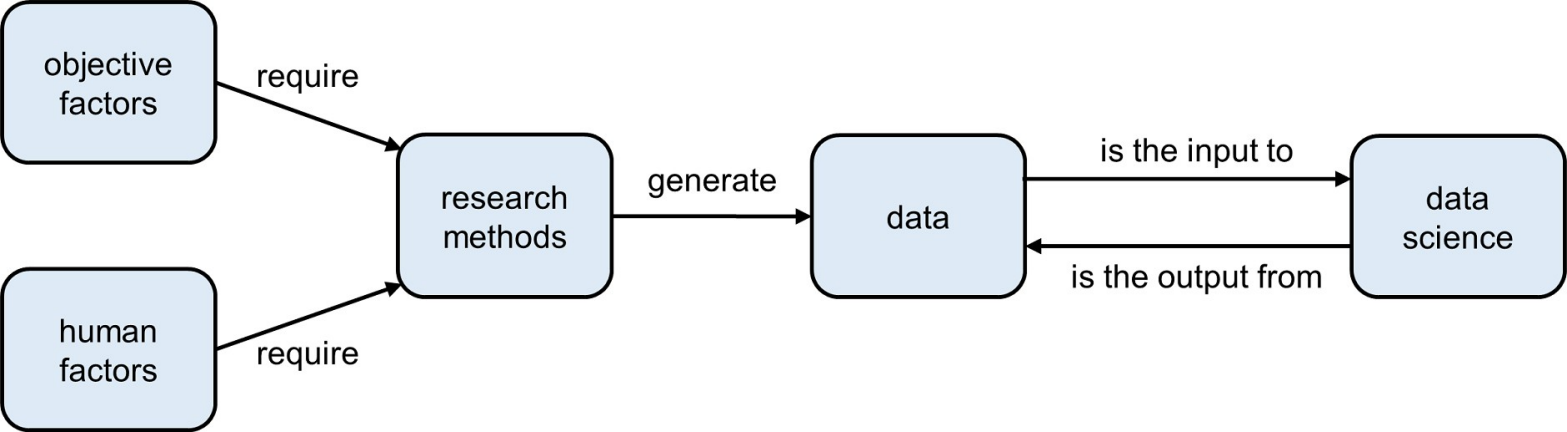
The framework of research content analysis.

Research content is much more complicated and domain-specific than bibliometric information. Although mature tools such as VOSviewer, CiteSpace and CitNetExplorer enable research content analysis as well, the fact that the embedded algorithms of such software distinguish only between the appearance of words or phrases but not the meaning of them could impair the detailed presentation of information. As a result, the outputs could be too general and lead to vague understanding. To solve this dilemma, this study attempts to reveal the research content of this interdisciplinary domain clearly by means of knowledge graph construction under a structured framework. Six steps were taken for this purpose:

- Framework construction and concept clarification. In accordance with knowledge graphs’ definition of multi-relational graphs composed of entities (nodes) and relations (edges) [15], Fig 1 illustrates the framework composed of entity types represented by rectangles and correlations represented by arrows. The five entity types stand for five research dimensions: (1) “objective factors” refer to the external elements of driving distraction, such as cellphone and roadside billboard; (2) “human factors” refer to the individual elements of driving distraction, such as age and gender difference of drivers; (3) “research methods” refer to the scientific methods for research conduction, like naturalistic driving study and driving simulation; (4) “data” refers to the information collected for analysis, like driving performance and eye activity; (5) “data science” refers to the processing methods to make raw data more meaningful, such as statistical means like logistic regression and artificial intelligence algorithms like deep learning. These entities are logically related: (1) “objective factors” and “human factors” are the starting point and purpose of studies; (2) “research methods” are chosen according to the requirement of “objective factors” or “human factors”; (3) “data” is generated by certain “research methods”; (4) “data science” takes in original “data” and regenerates more valuable “data”. Note that “objective factors” and “human factors” can be independent variables, dependent variables and even preconditions in studies. For instance, “mental workload” as a human factor can be selected as independent variable [28] or dependent variable [29], while “intersection” as an objective factor can be the precondition and does not have to be compared to other conditions [30]. Regardless of their roles in studies, these factors share the commonality of being the origins of research questions.

- Keyword identification and frequency accounting. Keywords depict the focus of a research domain. To obtain the keywords within the five dimensions and corresponding frequencies, *Author Keywords* and *Keywords Plus* of the retrieved documents were counted using Python 3.9. It should be noted that keywords with the same implication were mapped into one term. For example, “cellphone(s)”, “cell phone(s)”, “cellular phone(s)”, “mobile phone(s)”, “smartphone(s)”, “smart phone(s)” and “phone(s)” were all mapped into the term “cellphone”.
- Relationship extraction using Word2Vec. Relationships between keywords could be obtained through text mining with Natural Language Processing methods. To extract relationships between keywords, several operations were implemented on the original plain text file exported from *Web of Science Core Collection* with Python 3.9: (1) Titles and abstracts were extracted and lowercased, and functional words frequently appearing in abstracts such as “background” and “result(s)” were removed. (2) The NLTK (Natural Language Toolkit) package was employed for word tokenization (splitting textual data into tokens [31], the simple tokenization of splitting text into words adopted here), deletion of punctuations/numbers, removal of stop words and stemming. (3) Set phrases obtained by the counting of *Author Keywords* and *Keywords Plus* were converted into single words with ‘‘_” separating original words so as to ensure the completeness of minimum meaning units, and keywords with the same implication were mapped into one term. For example, “logist regress”, “logit regress” and “logit model” (“logistic regression”, “logit regression” and “logit model” before stemming) were all replaced with “logistic_regression”. (4) Word embedding was carried out using the Word2Vec model with the Genism package in Python 3.9. As a neural network-based deep-learning language model proposed by Google scholars, Word2Vec enables word representations in vector space and semantic similarity extractions among different words [32, 33]. Words in the preprocessed text were projected to a 300-dimensional vector space through this model, and a window of 10 words (the maximum distance between current word and prediction word in a sentence) was selected after fine-tuning. (5) Similarities of keywords were calculated based on the embedding vector of words from the Word2Vec model and false positives were removed manually.
- Visualization of all the keywords and relationships. To get an overview of the research content, the keywords and corresponding occurrence frequencies, as well as the values of relationships, were imported into the open-source software Gephi 0.9.2 for visualization [34], and the algorithm ForceAtlas2 was adopted for graph layout [35]. Note that “driving distraction” is omitted in the presentation of all these keywords. For instance, “passenger” implies drivers’ distraction problem with the presence of passengers.
- Visualization of the five dimensions. Keywords grouped according to entity types could serve the purpose of revealing each of the five dimensions in detail. To achieve this goal, the keywords obtained above were reviewed and classified into the five dimensions manually. Then keywords under each dimension, the occurrence frequencies and interrelationships were imported into the same software Gephi 0.9.2 for visualization, and the same algorithm ForceAtlas2 was adopted for graph layout.
- Visualization of relationships between dimensions. Among all the relationships, those between keywords under related dimensions in Fig 1 reveal the fundamentals of studies and so should be emphasized. To achieve this target, similarities of keywords under related dimensions were extracted and visualized in the form of heat maps.

## Results

### Results of bibliometric analysis

#### Yearly quantitative distribution of literature

On the whole, 3,790 publications and 42,220 citations were identified in the driving distraction domain during the period from 1990 to 2022. It should be noted that the numbers of 2022 do not indicate the whole year due to the retrieval time in February, 2022. The publication number of driving distraction in 2021 turns out to be 495 times that of 1990, 26 times that of 2000 and 5 times that of 2010. By contrast, the number of driving-related publications in 2021 turns out to be 103 times that of 1990, 11 times that of 2000 and 4 times that of 2010. It could be concluded that within the promising filed of driving research, increased attention has been drawn to driving distraction.

**Fig 2:**
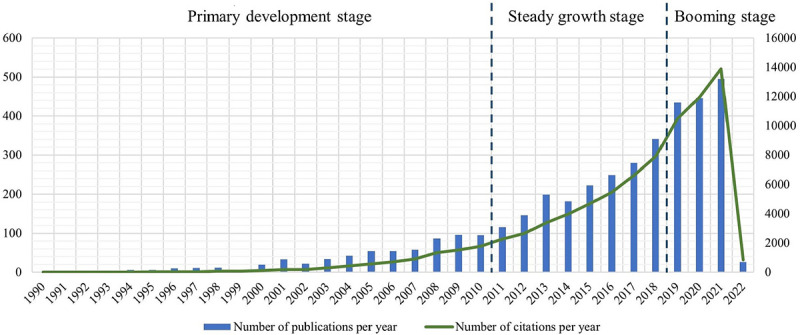
Annual distribution of publications and citations, 1990–2022.

The publication and citation numbers of the driving distraction domain are demonstrated in Fig 2. According to the explanatory graph, the evolution could be divided into 3 stages: the lengthy primary development stage (1990–2010), the intermediate steady growth stage (2011–2018) and the booming stage during the recent years (2019-now).

#### Major publication sources

**Fig 3:**
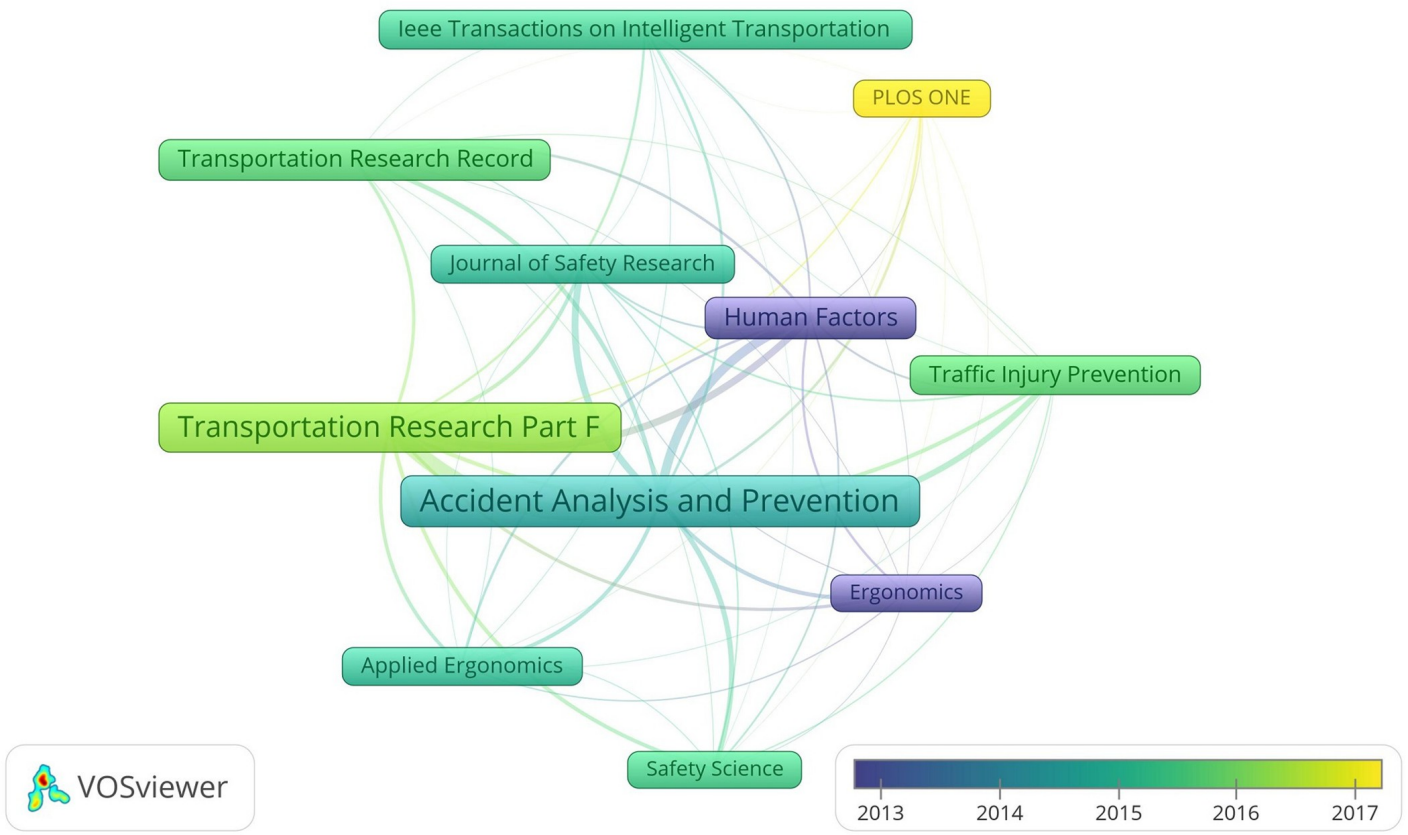
Major publication sources of documents.

Of the publication sources of the retrieved articles, 11 meeting the threshold of 50 documents and 500 citations are displayed in Fig 3. As shown in the graph, *Human Factors* and *Ergonomics* are among the earliest to pay attention to driving distraction. Although becoming concerned about this field later, *Accident Analysis and Prevention* and *Transportation Research Part F* appear as the center of the network at present, with 422 and 335 documents respectively. In recent years, the journal *PLOS ONE* turns as a new main participant of this domain. Although the network is concentrated, it’s worth noting that the journals in the network cover plenty of categories actually, like transportation, engineering, psychology, public health, social science, behavioral science, ergonomics and computer science.

#### Productive and influential countries/regions

**Fig 4:**
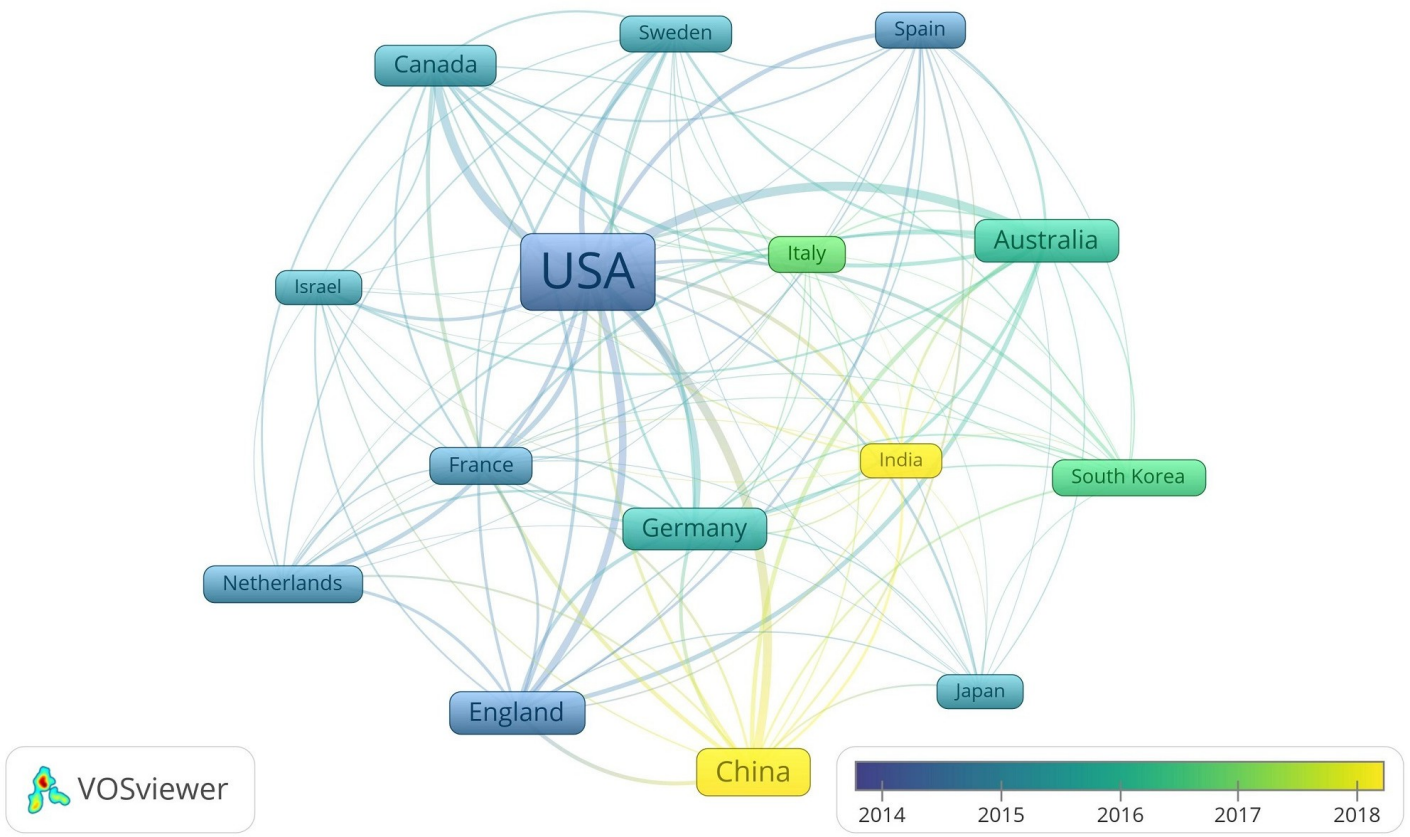
Geographical distribution of documents.

Of the countries/regions involved in the analysis, 15 meeting the threshold of 50 documents and 500 citations are displayed in Fig 4. As demonstrated in the graph, the USA is the absolute center of the geographical network, with 1,497 documents and 39,643 citations. Canada and European countries such as England and France are also active at this domain from the very beginning, whose documents hence enjoy relatively high average citations. Moreover, scholars in emerging Asian countries like China and India have also been making more exploration into this field over the past few years.

#### Productive and influential organizations

**Fig 5:**
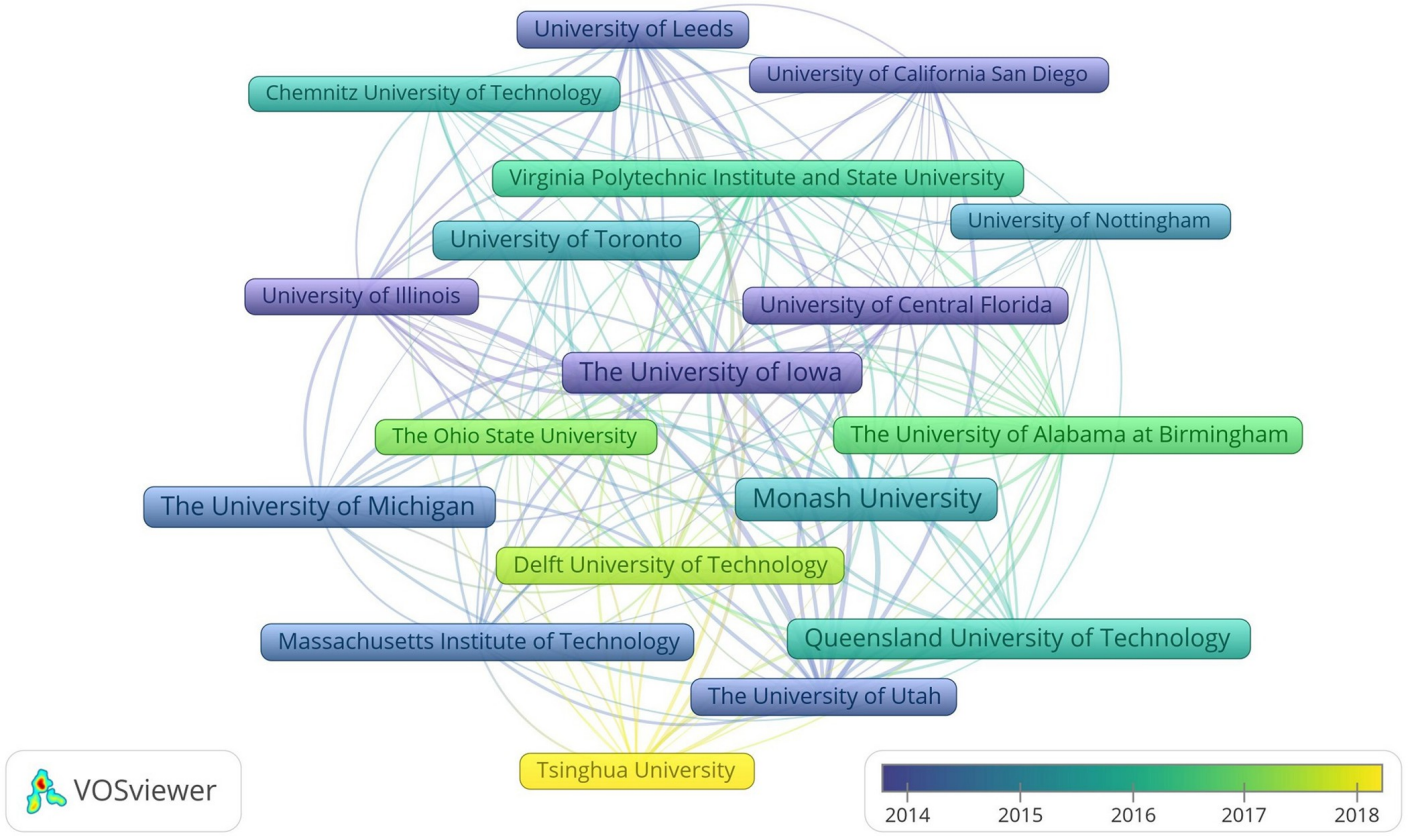
Distribution of documents among organizations.

Of the organizations concerned, 18 with the minimum number of 30 documents and 300 citations are exhibited in Fig 5. Being different from the document allocations among publication sources and countries/regions, the distribution among organizations is pretty decentralized, with only 74 publications belonging to the most productive one, Monash University. In relation to countries/regions, 10 of the 18 most productive and influential organizations are located across the USA, while the rest 8 ones are based in the Netherlands, England, Germany, Canada, Australia and China. In accordance with China as a new country player, Tsinghua University in China appears as a new organization participant.

#### Productive and influential researchers

**Fig 6:**
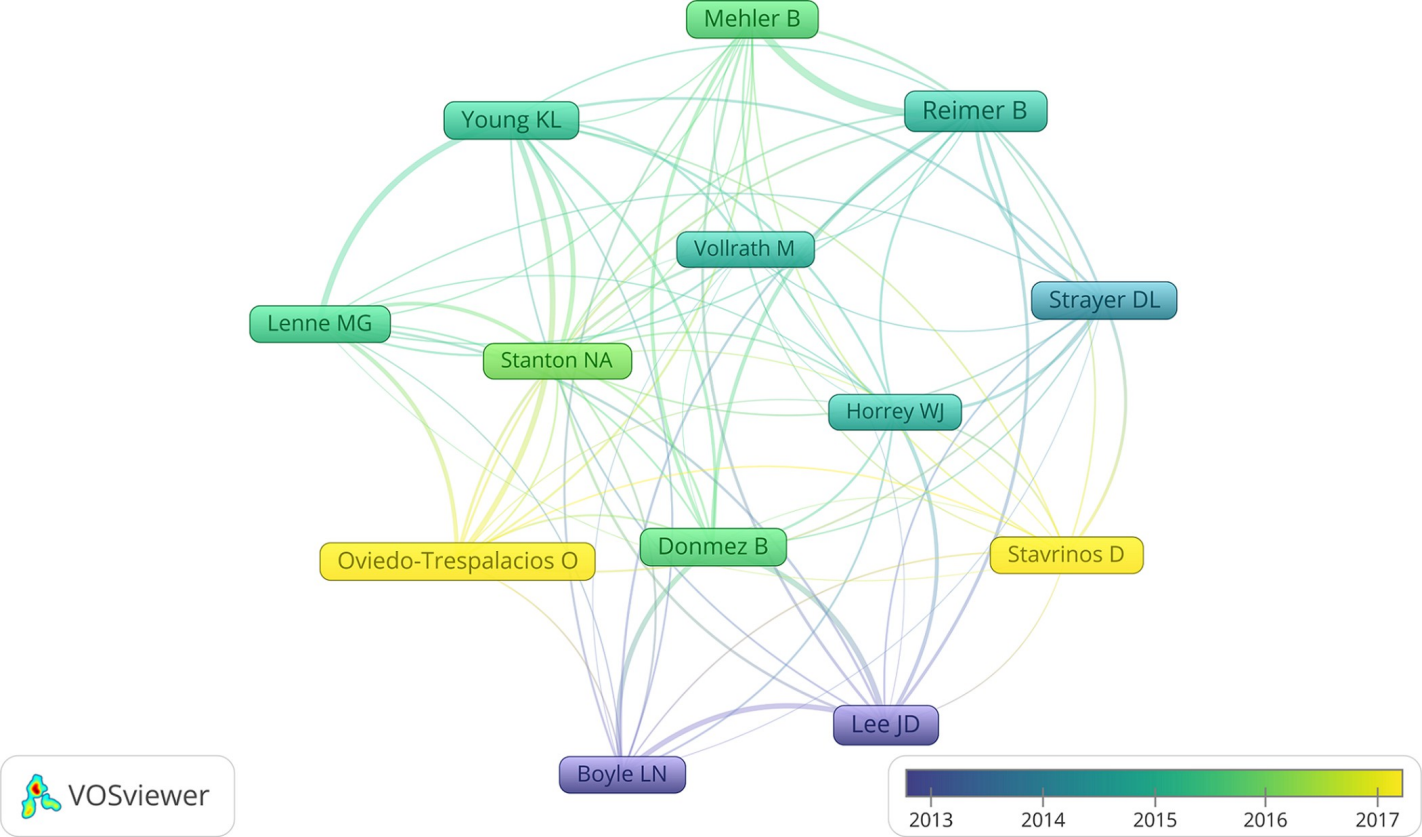
Main researchers in the driving distraction domain.

Of the researchers in this domain, 13 with at least 20 documents and 200 citations are exhibited in Fig 6. Reimer B turns out to be the most productive researcher with 37 documents, while Strayer DL appears as the most influential one with 1611 citations. Generally, all the researchers form a whole and there exists no separation. Clusters could be only identified when combining the graph and investigation into these researchers: Lee JD, Boyle LN, and Donmez B from the University of Iowa form a small cluster; Reimer B and Mehler B from the Massachusetts Institute of Technology are closely related academically; Young KL and Lenne MG from Monash University are close to each other.

## Results of research content analysis

### The domain of driving distraction as a whole

**Fig 7:**
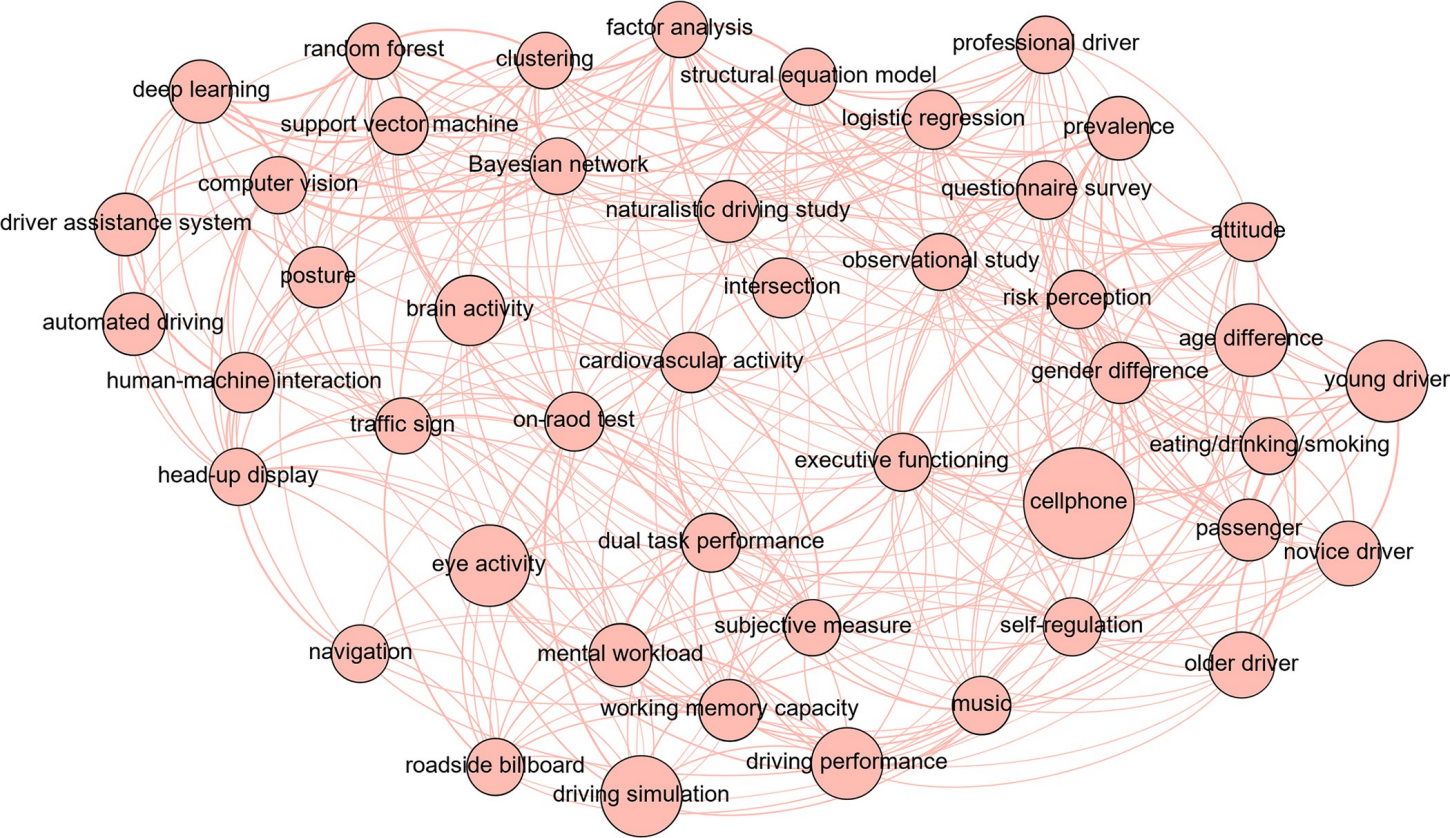
Visualization of all the keywords and relationships.

The knowledge graph composed of all the keywords and relationships shown in Fig 7 offers a general view of the driving distraction domain. As shown in the graph, “cellphone” turns out to be the most concerned issue and is related to entities like “passenger”, “eating/smoking/drinking”, “gender difference” and “age difference”, which all could be interpreted as classical and significant hotspots of the domain [36–38]. Algorithms like “deep learning”, “random forest” and “support vector machine” are grouped together, indicating that some studies may lay emphasis on comparison or integration of algorithms in application of the driving distraction domain [39]. More local connections could be identified as well. For example, the strong connection between “questionnaire survey” and “prevalence” reveals the adoption of questionnaire method to gain distraction prevalence data [40]. By and large, keywords are closely linked to form a whole, which confirms the inseparability of this domain but impairs understanding, especially for newcomers. Therefore, more structuralized clarification is necessary.

#### Main entities under each dimension

**Fig 8:**
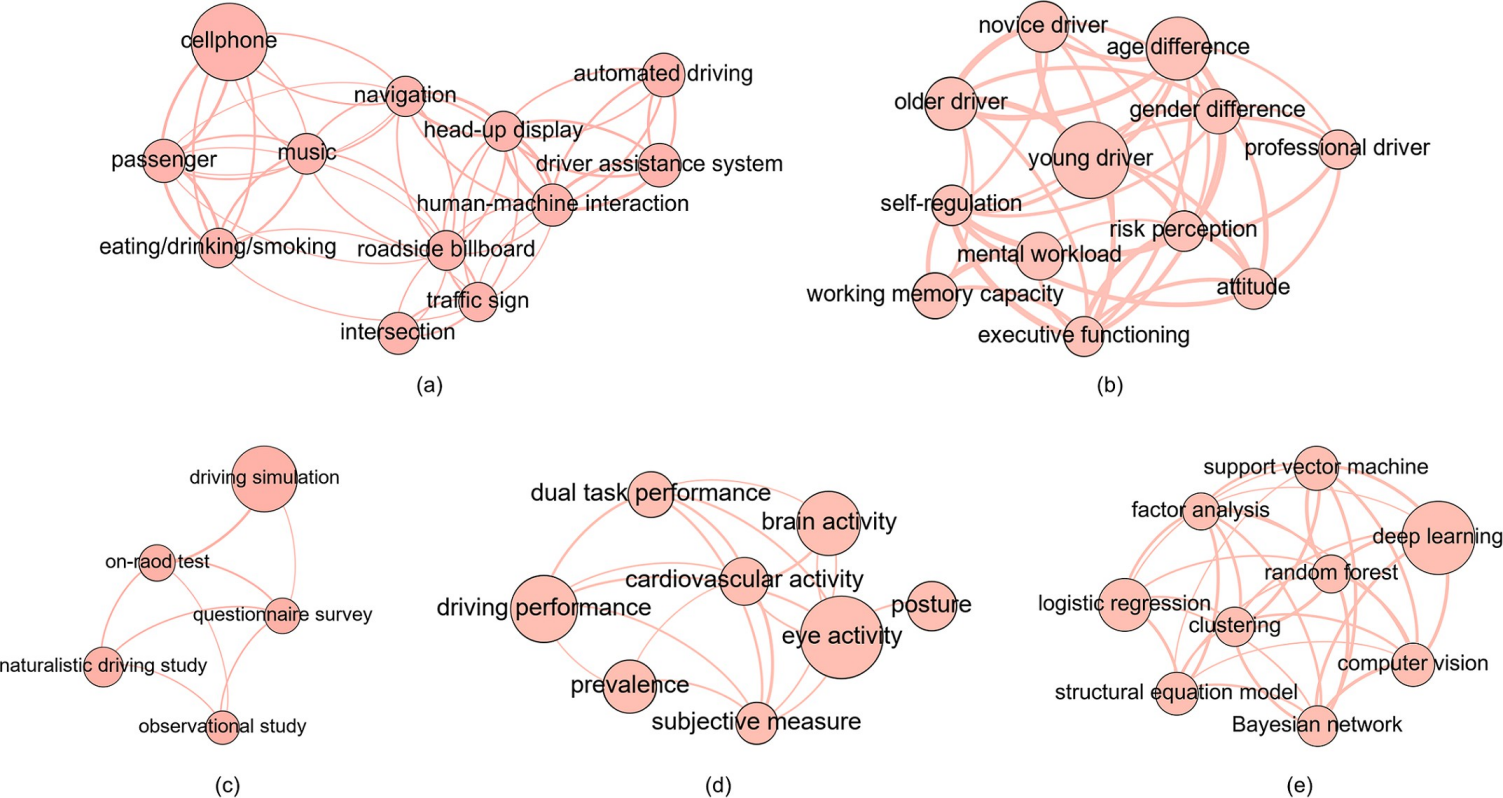
Visualization of the main entities under each dimension.

The framework of five dimensions illustrated in Fig 1 offers to structuralize research content of the driving distraction domain and facilitate understanding. Knowledge graphs for the five dimensions are demonstrated in Fig 8. In each subgraph, the nodes represent the main entities under each dimension, whose sizes indicate the occurrence frequencies, and the connecting lines reveal the degrees of closeness.

“Objective factors” shown in Fig 8(A) are the external starting point of driving distraction research. In vehicles, “cellphone”, conversation with “passenger”, “eating/smoking/drinking”, “music” and “navigation” are ordinary distractors [36, 37, 41, 42], “head-up display” has been proposed to reduce the distraction caused by conventional head-down displays in automobiles by keeping the glance on the road [43], and new “human-machine interaction” methods and designs have brought new vitality into distraction reduction [44, 45]. Outside of vehicles, “roadside billboard” and “traffic sign” are common distractors [6, 46, 47], while “intersection”, which could be subdivided into signalized and unsignalized ones, draws much attention with the complex traffic flow [48, 49]. “Driver assistance system” and “automated driving”, although leading to more freedom and less effort, could also provoke human drivers’ excessive engagement in non-driving tasks and cause accidents [50, 51].

“Human factors” shown in Fig 8(B) are the internal starting point of driving distraction research. “Age difference” regarding driving distraction has been widely proven, and “young driver”, “older driver” and “novice driver” are vulnerable groups [52]. As regards to “gender difference”, some studies suggested that males showed more confidence and engaged more in distracted driving [53], while others indicated that gender difference exclusively was not significant [54]. “Professional drivers” are a group of special concern due to the long driving time and tedious task in vehicles [55–57]. In addition, psychological factors, including mainly “working memory capacity” [58], “executive functioning” [59], “mental workload” [28], “risk perception” [60], “attitude” [60] and “self-regulation” [61], have been extensively discussed in relation to driving distraction. It should be mentioned that concepts of “working memory capacity”, “executive functioning” and “mental workload” are closely related in psychology, which all root in Working Memory (WM). The theory of WM assumes that *“a limited capacity system*, *which temporarily maintains and stores information*, *supports human thought processes by providing an interface between perception*, *long-term memory and action”* and brings about the term “working memory capacity”. The mainstream model of WM includes a central executive and three other components: the visuospatial sketchpad, the phonological loop and the episodic buffer. The central executive is linked to “executive functioning”, while the mental operations and efforts involved in these components produce “mental workload” [62–64].

“Research methods” required by scientific exploration into “objective factors” and “human factors” are demonstrated in Fig 8(C). Five main “research methods” are identified in this domain. “Driving simulation” remains as the main method from the beginning of this field up to now [65, 66]. In simulation, surrogate driving tasks such as LCT (lane change task) and BT (box task) could be adopted to mimic driving [67, 68], and tools like visual occlusion and n-back task could be employed for distraction imitation [69, 70]. “Naturalistic driving study” (NDS) provides insight into this issue under natural driving conditions [71]. “On-road test” is a middle way of simulation and NDS: similar to NDSs, participants engage in real driving tasks; similar to simulation, non-driving tasks are assigned by experimenters [72, 73]. Furthermore, the two epidemiological methods, “observational study” and “questionnaire survey”, reflect social behaviors and public opinions in time and thus support subsequent technological development, educational campaign and legislation [74, 75].

“Data”, the measure of driving distraction generated from “research methods”, is displayed in Fig 8(D). “Prevalence”, “driving performance” and “dual task performance” are direct evaluations [76, 77]. “Eye activity”, “brain activity”, “cardiovascular activity” and “posture” are all data depicting drivers’ real-time status. “Eye activity” could be obtained through eye tracker [78], camera [79] or electrooculogram (EOG) [80]. “Brain activity” is often acquired by electroencephalogram (EEG) [80] or functional near-infrared spectroscopy (fNIRS) [81]. “Cardiovascular activity” includes mainly heart rate and blood pressure. While heart rate and heart rate variability could be measured by electrocardiogram (ECG) or wearable devices, blood pressure could be exploited with blood pressure cuff applied to the upper arm and connected to monitor [82, 83]. And “posture” involving head, face, hand and body features is mainly recorded by camera [84]. Moreover, “subjective measure” of distracted drivers could be analyzed alone or together with the aforementioned objective data [85].

“Data science”, a hot and even sexy concept without precise definition actually [86], refers to the processing methods to make the original “data” meaningful for analysis in this study and is displayed in Fig 8(E). Statistical methods frequently adopted in this field include “factor analysis” [87], “structural equation model” [88] and “logistic regression” [76]. Traditional artificial intelligence approaches form another branch of “data science”, containing mainly “random forest” [89], “support vector machine” [90], “Bayesian network” [30] and “clustering” [91]. “Deep learning”, the promising direction of artificial intelligence, accounts for a large proportion of work in this field currently [80]. Meanwhile, the rise of “computer vision” has empowered distraction detection [10] and new human-machine interaction modes to reduce distraction like gesture interaction [92].

#### Relationships between related dimensions

**Fig 9:**
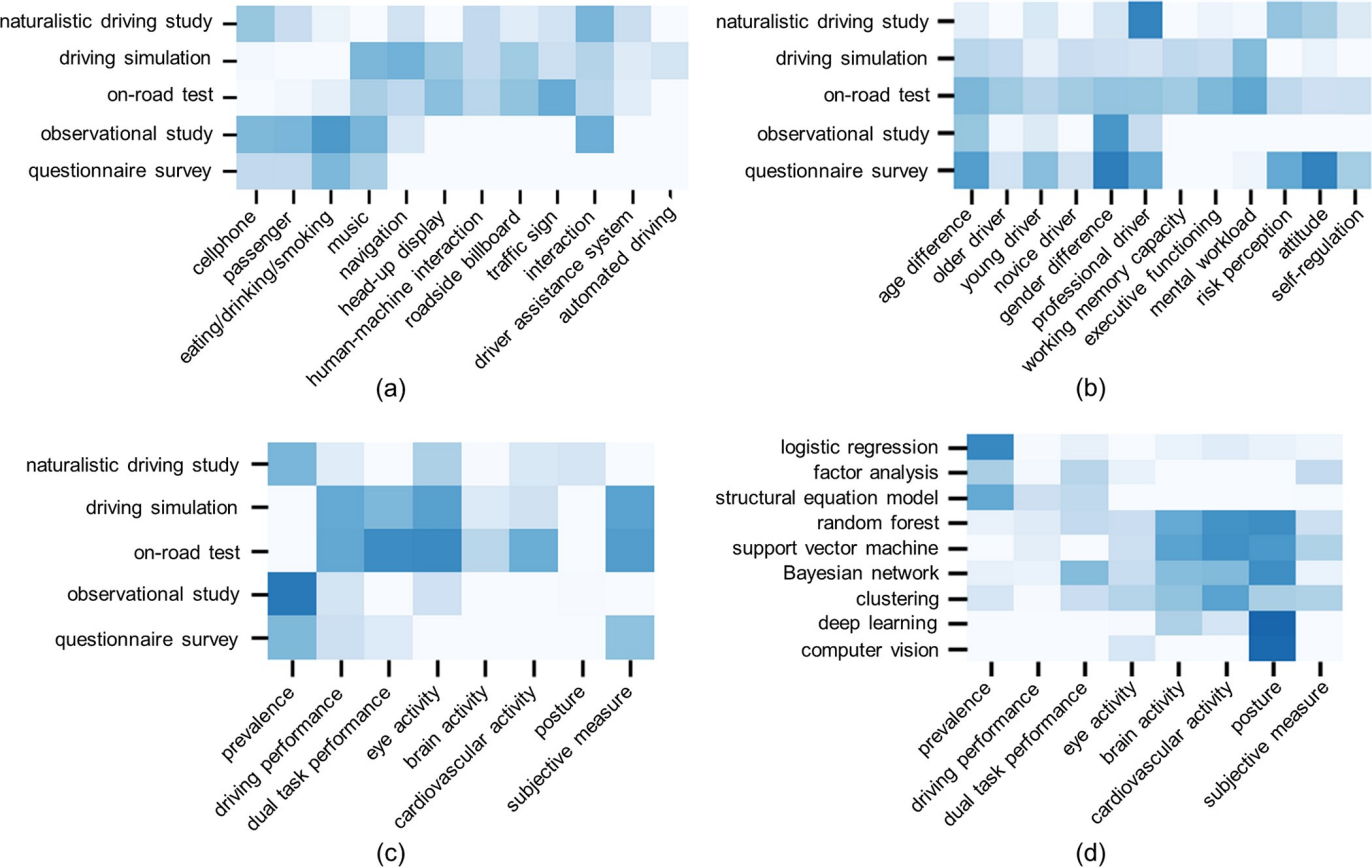
Visualization of the relationships between related dimensions.

The arrows in Fig 1 imply the logical relations of the five dimensions. Under this framework, entities in related dimensions are unevenly connected. Fig 9 reveals the connection strengths using heat maps.

Fig 9(A) and 9(B) display the application of “research methods” for exploration into “objective factors” and “human factors” respectively. According to the two subgraphs, “naturalistic driving study”, “driving simulation” and “on-road test” have been adopted to most factors, while the employment of “observational study” and “questionnaire survey” has been restricted to certain topics. Most factors turn out to be firmly related with more than one method except “automated driving”. And the current situation that “automated driving” is merely closely associated with “driving simulation” could be explained by the fact that it is still not available to the most public [93]. Yet the primary phase of automated driving still requires driver intervention, and the risk of excessive involvement in secondary tasks should be taken into consideration during the system development [51]. At the same time, certain research gaps still exist in this field. For instance, although “head-up display” has been introduced into the automotive field over two decades ago [66], there are still few naturalistic driving studies regarding its distraction impact.

Fig 9(C) demonstrates the association of “research methods” and “data” collected. “Driving performance” turns to be the only data type that is widely gathered by all the five methods. In observational studies and questionnaire surveys, driving performance is usually limited to crashes, near-crashes and traffic violations [94, 95]; in naturalistic driving studies, driving simulations and on-road tests, more sensors could be exploited to provide detailed data, such as driving speed, acceleration, headway, lane positioning, lateral acceleration and steering wheel angle [96–98]. Remarkably, collected mainly in naturalistic environments, “posture” data has been recorded to form public datasets to facilitate follow-up studies, like the 3MDAD dataset (Multiview, Multimodal and Multispectral Driver Action Dataset) and State Farm dataset [99, 100]. Some blanks in the heat map are decided by the nature of research method and data type. For example, “questionnaire survey” could never produce “brain activity” or “cardiovascular activity”. However, some gaps could be eliminated by future studies. For instance, “naturalistic driving study” and “brain activity” may be linked more closely with the application of wearable brain-computer interface devices in the driving research field [80].

Fig 9(D) shows the relationships between “data” and “data science”. According to this subgraph, statistical methods have mainly been employed for the procession of intuitive data like the prevalence of driving distraction, whereas traditional artificial intelligence approaches have been widely applied to nearly all kinds of data. Another observation is that “computer vision” and “deep learning” are both firmly correlated with “posture”, which should be attributed to the fact that the combination of “computer vision” and “deep learning” has contributed much to distraction detection utilizing the “posture” data. For instance, excellent deep learning models from the ImageNet Large Scale Visual Recognition Challenge (ILSVRC) in the computer vision field, such as AlexNet, GoogLeNet, ResNet50, VGG 16 and VGG 19, have been transferred to driving distraction detection [101, 102]. However, it should be mentioned that although the quantity of studies using deep learning is large (Fig 8(E)), the kinds of data treated with this method are limited (Fig 9(D)), which indicates the promising direction of applying deep learning to richer data types like driving performance.

## Discussion

### Trends of the driving distraction domain

The stage division demonstrated in Fig 2 reveals the evolution of this field. When combining bibliometric information with research content, the development trends could be more explicit.

During the primary development stage (1990–2010), literature of driving distraction started to emerge, and numbers of publications and citations fluctuated upward gradually, laying the foundation of this field. Classical topics like the impacts of hand-held/hands-free cellphone use and passenger presence were studied [36, 103], while vulnerable groups like young drivers and old drivers gained extensive attention from researchers [104]. And the simulation methods were widely adopted over this lengthy period [105, 106].

During the steady growth stage (2011–2018), the numbers of publications and citations increased smoothly. Large-scale naturalistic driving studies were conducted to understand driving distraction under natural conditions and brought about influential papers [71], and certain study programs with public accessibility like the Second Strategic Highway Research Program Naturalistic Driving Study (SHRP 2 NDS) have enabled follow-up studies worldwide till today [107–109]. Meanwhile, systematization was achieved with the clarification of relevant definitions [4] and the appearance of highly-cited meta-analyses [110, 111].

During the booming stage (2019-now), research in this field advanced to a new level of prosperity. This could be partly attributed to the new challenges and opportunities brought by progresses of driving automation systems [112]. In addition, distraction detection has become the hotspot over the years, and new directions have been proposed in this regard, like selecting features based on human factors domain knowledge to improve recognition accuracy [113], employing explainable artificial intelligence methods to promote comprehensibility of the recognition model and drivers [114] and adopting simple devices like smartphone sensors or wearable devices for data acquisition [115, 116].

The transition of major publication sources over time in Fig 3 indicates certain trend of this area as well. The early players of this field, *Human Factors* and *Ergonomics*, as the names themselves indicate, are devoted to the discipline of human factors/ergonomics (HFE), which is about all kinds of systems with people and allows interchangeable use of the two terms human factors and ergonomics actually [117]. Then journals concentrating on safety including *Accident Analysis and Prevention* and *Journal of safety research* paid much attention to driving distraction, and *Accident Analysis and Prevention* appears as the center of the graph with its emphasis on road safety currently [118]. Journals about transportation like *Transportation Research Part F* and *Transportation Research Record* turn out to be important contributors of relevant research as well. Furthermore, the appearance of the comprehensive journal *PLOS ONE* as the new participant implies that more importance is being attached to the interdisciplinary characteristic of this complex topic [119, 120], which then justifies the necessity of this study to discuss the driving distraction domain as a whole systematically.

The productive countries/regions, organizations and researchers shown in Figs 4–6 present the situation and developing trend across the world together. Research of driving distraction originated from North America and Europe, while other places of the world took part in gradually as well. It should be noted that although the USA appears as the center in the geographical distribution map, numerous organizations and researchers contribute to that together and there is no central organization or researcher in this domain actually. In recent years, the increasingly higher participation degree of developing countries like China and India brings new vitality to this field, especially when considering the large populations, complicated traffic conditions, improved economies and fast-developing social environments [121, 122].

### Significance of driving distraction research

Compared to other factors of road accidents like drunk driving or drug driving, which have been proven to be harmful and banned in most countries, driving distraction has only been partly prohibited by laws and remained controversial in the scientific community. The current situation could be attributed to the theoretical complexity of this issue, while future legislation and social norms could rely on the scientific understanding of this problem even more heavily.

The essence of distraction turns to be one source of the complexity and controversy concerning this issue. Essentially, driving distraction could be classified into “cognitive distraction”, “visual distraction” and “manual distraction”, which indicate “mind off the task”, “eyes off the road” and “hands off the wheel” respectively, and many behaviors in real driving could be attributed to a combination of two or three categories [123]. As regards to the risk of these three distractions, plenty of studies proved that all of them could exert negative impacts [124, 125], but there was also research suggesting that purely cognitive distraction did not increase accident rates obviously compared to normal driving [107, 126]. Taking the cellphone as an example, using handheld cellphones and texting messages while driving, which involve more than one kind of distraction, have been proven to damage driving by numerous studies and forbidden by laws in most countries, whereas the use of hands-free cellphones, which involves cognitive distraction only, is often legally allowed and has remained controversial among researchers [107, 127].

The intricacy of human beings adds to the complexity and controversy of this problem. The influence of the same distraction on different people may vary dramatically. Executive functioning is a typically distinguishable element. For people with high level of executive functioning, forcing them to concentrate on the single driving task may lead to boredom and fatigue, especially in simple environment, whereas secondary task engagement has the potential of increasing attention and improving driving performance [128]. Moreover, the nature of human beings is still to be discussed. Taking the mental workload as an example, contrary to traditional belief that lower workload leads to better performance, there has been study showing that transitions of mental workload could produce better primary and secondary task performance than duration of both high and low mental workload, which justifies appropriate secondary task engagement while driving [129]. At the same time, distinct characteristics of specific groups are not to be ignored in research as well. For instance, in terms of young or novice drivers, who are often the same group, limited driving experience, addiction to electronic devices and tendency of sensation seeking should be taken into consideration when discussing distraction-related accidents [12, 130]; in terms of older drivers, with the disadvantageous factor of physiological decline and the advantageous factor of abundant experience, tasks of different types and difficulties could exert entirely different influence [131, 132].

The significance of driving distraction research has been magnified by the progress of driving automation in recent years. According to the up-to-date division from SAE (Society of Automotive Engineers) and ISO (International Standardization Organization), the L1 (driver assistance) and L2 (partial driving automation) functions in widespread use nowadays serve the purpose of “driver support” merely, and the on-coming L3 (conditional driving automation) systems still require human attentions [93]. However, there may exists a “valley of degraded supervision” during the transition from “human driver” to “automated driver”, and undue disengagement from driving may lead to failure of human-machine cooperation and consequent accidents [50, 51, 133, 134]. Despite the risks of driving distraction, it is worth noting that forbidding distraction behaviors during automated driving totally may be not feasible. For one thing, driving automation indicates more freedom of drivers essentially and strict prohibition on non-driving tasks may adversely affect public acceptance of automation technology [135]. For another, when liberated from driving operations and prohibited from other tasks simultaneously at the automated wheel, the fatigue and sleepiness problem could be even more devastating than distraction [136, 137].

### Future directions

Several research directions could be identified according to the academic trend and practical need.

Firstly, research into the interaction of distraction and fatigue may generate strategies to improve overall safety. Fatigue is another well-concerned traffic accident factor in parallel with driving distraction [138]. Driving fatigue could be classified into sleep-related fatigue, active task-related fatigue and passive task-related fatigue [139]. Of the three types of fatigue, passive task-related fatigue in particular could interact with distraction. In fact, the possibility of introducing suitable secondary task to reduce passive fatigue has been validated by researchers [140]. Future research into the interaction mechanism of secondary tasks and fatigue may benefit the comprehensive understanding of human driver as a whole and the realization of optimal driver status.

Secondly, suitable secondary tasks could be customized according to automation levels, scenarios and people with different characteristics. It has been proven that whether secondary tasks exert positive or negative influence depends heavily on automation level [133], scenarios like complexity level of environment [128] and human characteristics [58]. Therefore, recommending secondary tasks like listening to music at the right time may be helpful both for driving safety and user satisfaction, and strategies to achieve this goal are to be obtained by future research. In addition, although the applications of laws regarding secondary tasks are the same under all conditions at present, future research may justify the necessity of allowing different secondary tasks under different circumstances and thus benefit the advances of laws.

Thirdly, meaningful features could be selected to improve distraction recognition. The development of data science has made the work of driving distraction detection possible even without knowledge about driving distraction, with the focus of detection being improving algorithms to achieve better performance on existing datasets [10]. Surely, these studies are of great significance. However, selection of indicators based on knowledge of the distraction domain should also be valued. For example, researchers have improved detection by incorporating glance features (percent road center, the standard deviation of gaze pitch, and yaw angles) that have been proved to be indicative of distraction in the human factor domain [113], while vehicle dynamics [141] and driver dynamics [116] have also shown potential in detection. Distraction recognition in the future could rely on the cooperation of the driving distraction, ergonomics and data science domain.

Fourthly, distraction in the context of L2+ automation deserves more attention. In the academic world, distraction issues of L2 and L3 functions have been addressed respectively [50, 51]. However, some automation functions on the market are actually between L2 and L3, like the NOA (Navigate on Autopilot) by Tesla, NOP (Navigate on Pilot) by NIO and NGP (Navigation Guided Pilot) by Xpeng, which all provide to drive automatically from one place to another place according to navigation. These are L2 functions nominally and drivers are required to put their hands on steering wheels during the use. However, different from basic L2 functions defined by SAE and ISO of executing lateral and longitudinal vehicle motion control [93], these functions’ ability to make decisions to change lanes so as to overtake or avoid obstacles means much to drivers and may change driver behavior. The misuse of these functions has been hot topics in practice, but little attention has been paid to them academically. Research to fill this gap is imperative to guide the practical transition from L2 to L3.

## Conclusions

In this study, knowledge graphs were constructed to understand the science domain of driving distraction from the two aspects of bibliometric information and research content.

For bibliometric information, according to the yearly quantitative distribution of literature, evolution of this domain could be divided into three stages, i.e., the primary development stage (1990–2010), the steady growth stage (2011–2018) and booming stage (2019-now), which are accompanied by transition of research content. Meanwhile, knowledge graphs from Vosviewer indicate certain trends of this field as well. The publication source graph reveals the interdisciplinary characteristic of this issue and the necessity of systematic consideration. The graph of countries/regions implies the shift of geographical distribution from developed countries to the whole world. And graphs of organizations and researchers show the decentralized distribution and extensive connections.

For research content, which is more complicated and domain-specific, a new framework consisting of five dimensions was established and knowledge graphs were constructed on this basis. “Objective factors” and “human factors” are the origins of research, represented by the common distractor cellphone and young/novice drivers’ issue respectively. “Research methods” required by objective or human factors include mainly driving simulation, naturalistic driving study, on-road test and the two epidemiological methods of observational study and questionnaire survey. “Data” collected from these methods is diverse, varying from driving or dual task performance to drivers’ eye or brain activity. “Data science” to make data more meaningful involves mainly statistical methods, traditional artificial intelligence methods, deep learning and computer vision. Moreover, research gaps and potential directions have been identified and clarified.

Driving distraction has remained a major factor of road accidents with special complexity and controversy, which has been manifesting even more clearly in the new era of driving automation. Research into this issue serves as the foundation of legislation for accident reduction practically and promotes the understanding of humankind theoretically. More studies are expected to fill the blanks and advance the progress of this field.

## Supporting information

S1 File Part one of the data. (TXT)

S2 File Part two of the data. (TXT)

S3 File Part three of the data. (TXT)
